# Physicochemical property consensus sequences for functional analysis, design of multivalent antigens and targeted antivirals

**DOI:** 10.1186/1471-2105-13-S13-S9

**Published:** 2012-08-24

**Authors:** Catherine H Schein, David M Bowen, Jessica A Lewis, Kyung Choi, Aniko Paul, Gerbrand J van der Heden van Noort, Wenzhe Lu, Dmitri V Filippov

**Affiliations:** 1 Institute for Translational Sciences, Computational Biology, Sealy Center for Structural Biology and Molecular Biophysics, University of Texas Medical Branch, Texas 77555–0857, USA; 2 Departments of Biochemistry and Molecular Biology, University of Texas Medical Branch, Texas 77555–0857, USA; 3 Microbiology and Immunology, University of Texas Medical Branch, Texas 77555–0857, USA; 4 Department of Pathology, University of Texas Medical Branch, Galveston, Texas 77555–0857, USA; 5 Dept. of Molecular Genetics and Microbiology, Stony Brook University, Stony Brook, NY 11790, USA; 6 Leiden Institute of Chemistry, Leiden University, PO Box 9502, 2300 RA Leiden, The Netherlands

## Abstract

**Background:**

Analysis of large sets of biological sequence data from related strains or organisms is complicated by superficial redundancy in the set, which may contain many members that are identical except at one or two positions. Thus a new method, based on deriving physicochemical property (PCP)-consensus sequences, was tested for its ability to generate reference sequences and distinguish functionally significant changes from background variability.

**Methods:**

The PCP consensus program was used to automatically derive consensus sequences starting from sequence alignments of proteins from Flaviviruses (from the Flavitrack database) and human enteroviruses, using a five dimensional set of Eigenvectors that summarize over 200 different scalar values for the PCPs of the amino acids. A PCP-consensus protein of a Dengue virus envelope protein was produced recombinantly and tested for its ability to bind antibodies to strains using ELISA.

**Results:**

PCP-consensus sequences of the flavivirus family could be used to classify them into five discrete groups and distinguish areas of the envelope proteins that correlate with host specificity and disease type. A multivalent Dengue virus antigen was designed and shown to bind antibodies against all four DENV types. A consensus enteroviral VPg protein had the same distinctive high pKa as wild type proteins and was recognized by two different polymerases.

**Conclusions:**

The process for deriving PCP-consensus sequences for any group of aligned similar sequences, has been validated for sequences with up to 50% diversity. Ongoing projects have shown that the method identifies residues that significantly alter PCPs at a given position, and might thus cause changes in function or immunogenicity. Other potential applications include deriving target proteins for drug design and diagnostic kits.

## Background

The most useful information one can glean from aligned sequences of proteins is first, the absolutely conserved residues, which are usually those that maintain the structure of the protein or are vital for function. The pattern, or profile of conserved residues in an alignment of a protein type can be used to identify proteins in the same group, that may have a similar structure [1-4]. In addition, the variance within the sequences, which may occur at specific positions due to random variation (i.e., in RNA viruses, an error prone polymerase), can also indicate a functional change. It is thus important to be able to separate background variation, which, in our approach, is assumed to cause little change in the physicochemical properties (PCPs) at a position, from those that alter these properties sufficiently to lead to a variance in protein function or immunogenicity [5-8].

**Figure 1:**
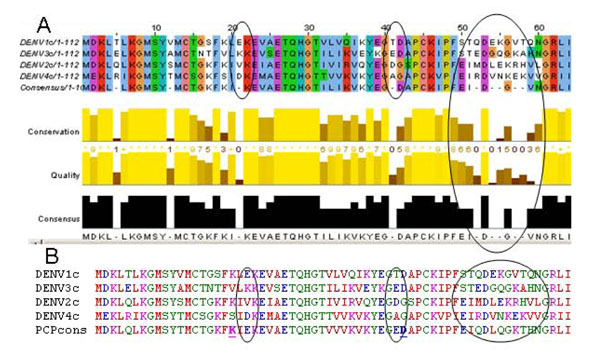
**Dealing with variable positions in aligned sequences.** A consensus that chooses only the most frequently occurring amino acid at a given column of an alignment (e.g., that from the Jalview applet of Clustal W) is best for indicating highly conserved residues, but does not give any consensus value for variable positions (such as those circled). B) A PCP-consensus for the same sequences selects the amino acid that is most similar in properties to all others in the set.

Very large alignments present intrinsic problems in discriminating residue conservation or patterns of variance, and require special software even to view them. Another problem in dealing with biological datasets, such as the many Flavivirus sequences we have collected within the Flavitrack database [9,10], is that they often have a pronounced bias due to unequal distribution, which can arise from non-uniform sampling [11]. For example, one may have many closely related sequences from one epidemic, where serious infections occurred, but few from the intervening years, when most infections had a less lethal phenotype. Conventional methods for calculating consensus sequences assume an unbiased data set, and typically calculate only the most common amino acid in a column [12]. An example of such a consensus (Figure 1A) shows that while it provides useful information on the degree of conservation of the amino acids in aligned sequences, it cannot suggest a rational choice of amino acid at highly variant positions. Profiling methods [13-15] based on amino acid scoring matrices can also be used to obtain a consensus sequence, but these are primarily designed to detect distantly related members of a set of proteins.

Here we show applications of a general method to calculate physicochemical property (PCP)-consensus sequences. The method is designed to filter noise due to random amino-acid variations within strains or subtypes from more significant variation. We first modeled PCP-consensus sequences for several proteins, and showed that they were stable after minimization with our FANTOM program. We have also produced several PCP-consensus proteins from synthetic gene sequences in *E. coli* and tested their ability to be recognized enzymatically and immunologically. As discussed below, PCP-consensus sequences have many uses, in sequence classification, epitope comparison, in defining multivalent sequences as immunogens for vaccine use, and for defining targets for multivalent drug design.

## Methods

### Deriving PCP consensus sequences

Our method assumes that one has a high quality alignment, of any number *N* of sequences with a maximum length L. Choosing an appropriate sequence grouping is a chicken/ egg problem, and will be discussed in more detail below. Multiple sequence alignments were generated with Clustalw 2.0.3[16], or MUSCLE [17,18], for very large alignments, using default parameters. It is best to check such large alignments for inappropriate gapping. Although there are statistical methods to do this [19], for the purposes of this early validation work, we have chosen homologous proteins where a representative protein structure is known, and sequence groupings that have more than 50% identity. This allows us to check that any gapping is consistent with secondary structure elements, and conservation of disulfide bonds and salt bridges.

**Figure 2:**
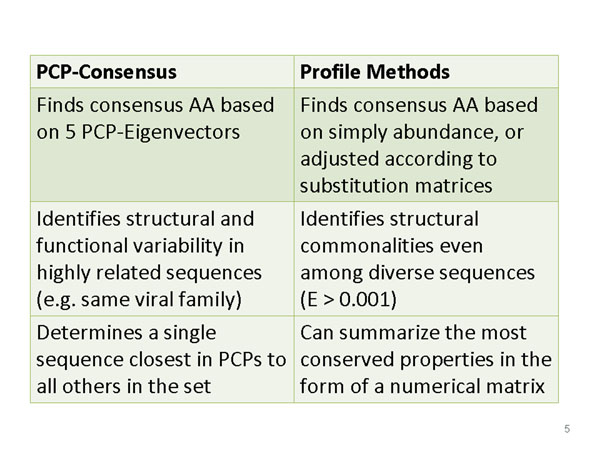
Comparing the PCP-consensus method and its uses to profiling methods and their applications.

Figure 2 compares the differences in the purpose and method for obtaining a PCP consensus sequence with those of profiling methods. To obtain PCP-consensus sequences, at each position of the multiple alignment, one amino acid is chosen that best approximates the average value of the PCPs. The PCPs of the 20 amino acids are defined by a set of numerical descriptors, 5 Eigenvectors obtained by multidimensional scaling of over 200 unique property measurements [20]. Each amino acid can be discriminated from all the others as points in a five dimensional space, where the five dimensions, the first 5 eigenvectors, roughly correspond to hydrophobicity/hydrophilicity (E1); size (E2); alpha-helix propensity (E3); the property E4 is related to the partial specific volume, number of codons and relative abundance of the amino acids; and E5 correlates weakly with beta-strand propensity [20]. This 5-dimensional approach to similarity allows one to calculate a true consensus amino acid, i.e., the one closest in its PCPs to all others in the column, even at very variable positions. Given an amino acid alignment, the program selects an amino acid that is closest in the property space to the average values for all the other amino acids at each position. First, the average value of each of the 5 PCP- vectors *p*=E1,..,E5 is determined at each column, effectively turning an alignment of *N* sequences of maximum length L into a 5xL matrix:(1)

where *N* is the number of amino acids in the given column of the alignment; and , the value for the relevant p eigenvector for the jth amino acid. Then the consensus amino acid (*A_a_*) is chosen from those occurring naturally at that position with the least Euclidean distance from the average:(2)

The alignment independent scale factors *b_p_* were calculated so that vector values with higher relative entropies at a given column would be more significant, and were calculated as described elsewhere [9].

For very variable positions or highly biased datasets, the amino acids that naturally occur at each position can be used one time, without regard to their rate of occurrence in the column, to calculate the average values of the 5 property vectors. In that case, equation 2 can still be used, and the chosen “consensus” amino acid is simply that closest in its physical properties to all the naturally occurring amino acids. Other possibilities for dealing with bias in the data set, such as selective sequence weighting can also be used [21] to determine the property averaging method. This is an area for further study, as a completely mathematical solution for all situations is probably not possible.

It should be stressed that biological findings can be incorporated at any point in this process, in distinguishing sequences that have specific properties. Bioinformaticians should be aware that sequences grouped according to a biological assay may or may not correlate with distinctive genotypes. For example, the four types of Dengue viruses (DENV1-4), first characterized by Sabin in the early fifties based on immunological reactivity [22], segregate rather cleanly into four distinct genotypes (see below). However, human enteroviruses (HEV), designated Coxsackie virus A or B based on the type of paralysis they caused in newborn mice, did not separate neatly into two distinct sequence groups [23]. While we have relied on the strain designations in the NCBI for Flaviviruses, other useful data that should be part of the functional annotation (such as lethality) is often not specified by those providing the sequences to NCBI.

**Models of PCP-consensus sequences** were prepared with our MPACK modeling suite [24-27] using the crystal structure of the DENV-2 protein (1OAN.pdb) [28].

## Results and discussion

### PCP-consensus sequences using Flavitrack entries

There are many potential uses for PCP-consensus sequences in virology, for example in classifying strains, identifying functional alterations [29], and in designing novel, multivalent antigens for vaccines and diagnostics. Here we will show some applications based on data stored in our Flavitrack database (http://carnot.utmb.edu/flavitrack), which is a compendium of annotated Flavivirus sequences [9,10]. Flaviviruses (FV), which include yellow fever (YFV), DENV, and West Nile viruses (WNV), are important human and animal pathogens which typically require insect vectors to infect mammalian hosts [30-35]. While mosquito control can be effective, antiviral agents and wide-spectrum vaccines are being sought to protect those in endemic areas [36-43]. To design effective vaccines, the areas of the viral proteins required for virus function or infectivity should be targeted by antibodies. Flaviviruses are variable, with many sequence variants found even in single virus isolates from the same patient, so-called “quasispecies” [44]. However, when catalogued, the strains appear redundant from a mathematical standpoint, with interstrain diversity occurring at fewer than 1% of positions. While much of this variation is neutral for phenotype, even a single point mutation can greatly alter the immunogenicity of the envelope protein or alter virus entry [38,40,45-47]. Recognizing such function-altering amino acid substitutions is important for designing vaccines that will protect against many Flaviviruses simultaneously, and entry inhibitors.

Our first programs for analyzing the sequences in Flavitrack attempted to highlight all variation in the aligned sequences in a fashion suitable for conventional visual scanning of the data. These first attempts illustrated the need for unbiased data reduction: an alignment of 928 sequences (Flavitrack ca. 2009) covered dozens of pages of paper. Even at the smallest possible type (a microtext version of the database sequences provided to us by Reiner Eschbach’s group at Xerox), no screen setting was adequate to view more than a small part of the data. Also for the purposes of determining variation, we needed a rational mean sequence to compare sequences. Other intrinsic problems in the data were the non-random sequence distribution, with many more sequences available for certain mosquito-borne viruses (WNV and DENV) than for any of the tick-borne or no-known-vector (NKV) groups.

### PCP-consensus reference strains

**Figure 3:**
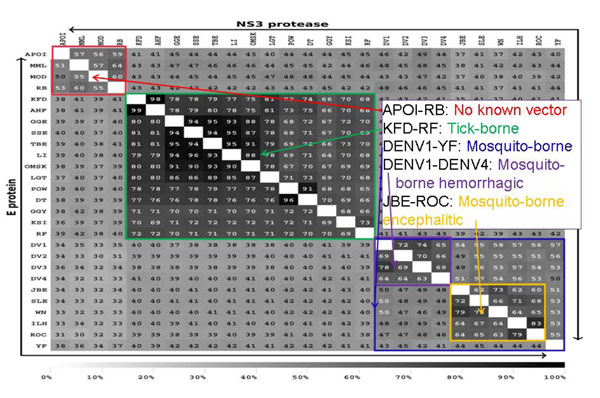
**PCP-consensus sequences distinguish 5 groups of Flaviviruses**. The PCP- consensus sequences of two viral proteins, the envelope (E) and NS3 protease, of 37 different Flavivirus strains derived from 928 annotated entries in Flavitrack [48], separate into five distinct clusters, based on their percent identity to one another.

The traditional Flavivirus group reference strains are historical isolates with defined immunological properties, often predating the genome era, and not chosen to best represent a consensus genotype. These strains may have been passaged many times in the lab setting, a process that could result in a sequence quite distant from the original or any subsequently isolated wild type strain. Now that direct PCR sequencing from the original isolate is possible, a more relevant methodology would be to use a series of PCP-consensus reference strains, as long as these were shown to correlate with serotype data or other biological assays. We began by creating PCP-consensus sequences for each group of Flaviviruses, where the groups were defined according to the strain designations in the NCBI headers for the annotated sequences in Flavitrack [48]. A series of 37 PCP-consensus strains for the most common groups of Flaviviruses was derived (Figure 3), which allowed comparison of the overall properties of the individual virus types and separated the Flaviviruses, based on two different proteins, into five discrete groupings. We suggest that these can be viewed as reference strains for grouping (or profiling [49]) novel Flavivirus isolates, obtained for example, from sentinel screening of mosquitoes [50].

Proper grouping of virus isolates has meaning beyond mere nomenclature: comparing these consensus sequences, we could discriminate residue changes that fell outside the expected group variance. The comparison highlighted insertions and deletions that correlated with whether a species was carried by mosquitoes or ticks, and even with the type of disease (encephalitic vs. haemolytic) resulting from human infection [48]. Additional uses of classification are to detect when a strain that appeared to be adapted to growth only in mosquitoes or bats contained key substitutions that might indicate human cross-over potential.

### A multivalent PCP-consensus DENV antigen

**Figure 4:**
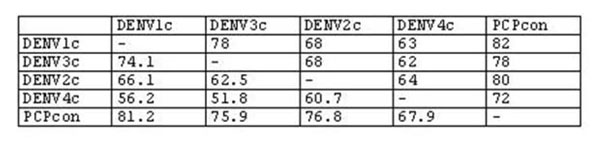
**Identity matrix for four individual DENV consensus strains and a multivalent PCP consensus derived from them.** The matrix shows the inter-sequence Clustal W scores (top) or % Identity (bottom) for PCP-consensus sequences for the envelope protein of each of the four DENV types, and a PCP consensus prepared from these four individual consensus sequences. The overall PCP-consensus is about equidistant from the four consensus sequences, using either metric for similarity. Note the four distinct genotypes, that DENV 1 and 3 are closer to each other than to DENV2, and that DENV4’s sequence is distant from the other 3 types.

**Figure 5:**
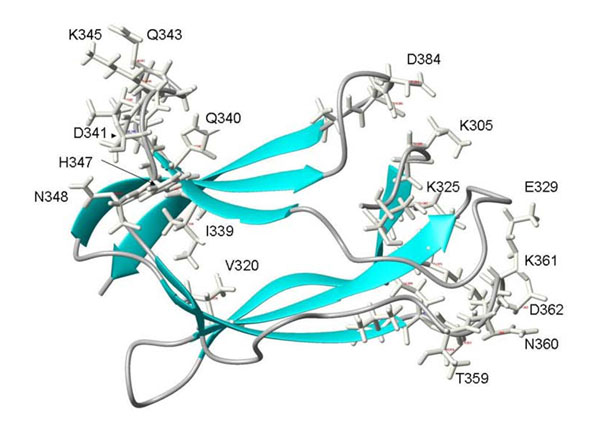
**Variable positions in a PCP-consensus DENV antigen.** A model of a PCP-consensus EdomIII DENV antigen is shown in ribbon format, with the sidechains where maximum variation occurs shown in stick format. Labels on the outermost residues are shown for orientation, and to illustrate that the type specific epitope surfaces are not linearly encoded in the amino acid sequence.

**Figure 6:**
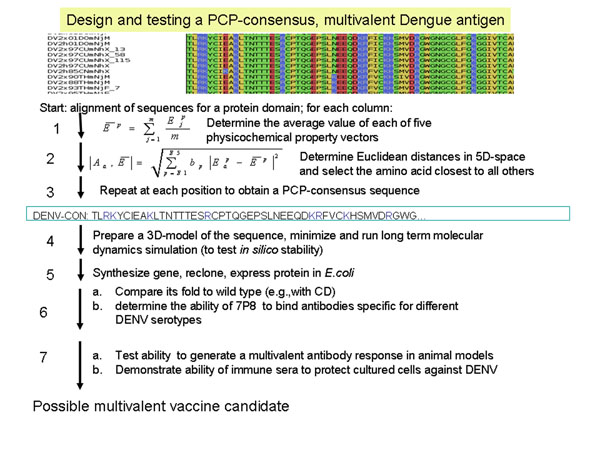
**Deriving a multivalent DENV antigen for testing in animals.** Designing, producing and testing a multivalent DENV antigen for vaccine use. The protein was able to bind antibodies generated against all four types of DENV, and induced antibodies in a rabbit that recognized wild type proteins from all four types of DENV.

Understanding the variation between viral types is particularly important for DENV, as it has been shown that reinfection of a person carrying antibodies against one DENV immunotype with a different type can result in Dengue hemorrhagic fever (DHF), a severe disease that requires proper medical support. In 2010, an epidemic in Brazil caused over a million documented cases, with about 600 deaths (many of which were young children). Billions of people throughout the world are at risk for DENV [43,51-55]. We approached this problem by generating a PCP-consensus of the representative envelope proteins for the four different types of Dengue virus (DENV1-4). This consensus was close to each type and thus represented a good mean (Figure 4). This was especially clear for the outlier, DENV-4, which was lowest in absolute identity to all the other three DENV types and is the hardest to neutralize with antibodies generated by infection with DENV-1,2 or 3. Figure 5 shows a portion of the consensus envelope protein of Dengue, with residues of maximum variation highlighted. Optimal choice of residues in variable areas of the viral strains can guide the design of multivalent vaccines and inhibitors. Figure 6 shows how this sequence was further developed for testing in animals. The recombinant protein, after optimization to reflect the DENV4 outlier, bound antibodies to all four DENV types [56] and preliminary work has shown that inoculation of this protein generates multivalent antibodies in rabbits and mice that neutralized all four DENV strains (data not shown).

### PCP-consensus VPg for designing multivalent inhibitors of human enteroviruses

To further illustrate the potential uses of the method, we designed and produced a PCP-consensus “viral peptide linked to the genome” (VPg) for the human enteroviruses (HEV), which include polioviruses (PV), Coxsackie viruses A and B (CVA and CVB), and Echovirus. To initiate RNA synthesis, HEV polymerases (3D-pol) uridylylate a conserved tyrosine residue in the 22 amino acid long VPg to form VPgpU. We have determined the NMR structure of poliovirus type 1 (PV1)-VPg and PV1-VPgpU and shown that uridylylation stabilized the 3D-structure of the peptide, which is probably necessary for VPgpU to serve as a precursor for RNA synthesis [57-59]. As this reaction is not found in normal cells, it is a target for antiviral drug design [57]. To develop a multivalent target VPg suitable for designing inhibitors against all HEV, the sequences of 33 unique HEV-VPgs were aligned and a PCP consensus protein, VPg-cons, was prepared with our automatic program. Although only about 50% of the amino acids were conserved completely the selected VPgs, the calculated pKa values of all of them were exactly 10.46, suggesting the peptide must be very basic in order to function (this is consistent with our NMR structures, which shows the basic, essential side chain of Arg17 very close to the phosphates of the coupled UMP). The PCP-consensus VPg, which is not identical to any naturally encoded sequence, had the same calculated pKa of 10.46. This illustrates that the consensus represents conserved physicochemical parameters of a sequence set. Both the PCP-consensus HEV-VPg and HEV-VPgpU were prepared synthetically [60,61]. The HEV-VPg can be uridylylated by both the PV1- and CVA24-RNA-polymerases as well or better than the wild type VPg encoded in their respective genomes. Thus the PCP-consensus VPg represents the conserved properties of HEV wild type VPgs, and functions in a multivalent manner. Further study of the structure of the HEV-VPgpU should aid in deriving a general mechanism for uridylylation. Inhibitors based on the consensus HEV sequence should be multivalent, and prevent replication of all HEVs.

## Conclusions

Defining PCP-consensus sequences can aid in analysis of large sequence datasets. The calculation method, based on a previously validated 5D-vector scale for the physicochemical properties of the amino acids, is straightforward, once a suitable alignment of related sequences is obtained. Having a rational consensus allows one to distinguish residue variations that significantly alter the properties at a given position. The method is thus suitable for application to many types of bioinformatics data.

The usefulness of the methodology in virology was demonstrated in two practical applications. A multivalent, PCP-consensus DENV vaccine candidate was designed, produced, and shown to bind antibodies against all four types of DENV. Also, a consensus HEV-VPg has similar properties, particularly pKa, conserved in wild type VPgs, and was uridylylated by two different HEV polymerases. This validated method should find application in many practical areas of virology and other areas of biology.

## Competing interests

The authors declare that they have no competing interests.

## Authors' contributions

CH developed the PCP-consensus method and participated in all the experiments described, as well as writing the paper. DB and JL prepared the PCP-consensus DENV antigens and did ELISA tests for binding. KC prepared polymerases for the VPg assay; AP did assays for uridylylation of the consensus VPg with two polymerases; WL (graduate student) did PCP-consensus calculations; GHN and DF prepared PCP-consensus VPg and VpgpU peptides.
